# 3D analysis of smile transformation in patients with class III deformities following orthognathic surgery: a stereophotogrammetric study: Original Article

**DOI:** 10.1007/s00784-026-06748-4

**Published:** 2026-01-20

**Authors:** Alaz Enez, Nur Altıparmak, Berat Serdar Akdeniz, Sıdıka Sinem Akdeniz

**Affiliations:** 1 Faculty of Dentistry Department of Oral and Maxillofacial Surgery, Baskent University, Cankaya, 06490 Ankara Turkey; 2 Gulhane Faculty of Dental Medicine, Department of Orthodontics, University of Health Sciences, Yenimahalle, 06290 Ankara Turkey; 3 NUO Dental Clinics, Private Practice, Çankaya, 06530 Ankara Turkey

**Keywords:** Three-dimensional (3D) stereophotogrammetry, Orthognathic surgery, Smile parameters, Buccal-corridor ratio

## Abstract

**Objectives:**

Smile aesthetics are crucial in orthodontic and orthognathic planning, particularly for patients with dentofacial deformities seeking enhanced facial harmony. This study evaluated changes in smile parameters of patients with class III deformities before and after orthognathic surgery using 3D stereophotogrammetry.

**Materials and methods:**

Conducted at Başkent University Oral and Maxillofacial Surgery Department, this retrospective investigation comprised 21 patients with class III skeletal deformities who underwent bimaxillary orthognathic surgery. Standardized social-smile photographs and 3D facial scans were obtained preoperatively and at an average 8-month follow-up. Smile parameters, including buccal-corridor ratio, upper-lip length, inter-commissural width, lip thickness, lip asymmetry and volumetric measures were reported. Preoperative and postoperative differences were analyzed with paired T-tests or Wilcoxon signed-rank tests (*p* < 0.05), 95% confidence intervals, and Cohen’s d for paired samples.

**Results:**

Mean follow-up was approximately 8 months. False-discovery-rate correction identified one significant change: buccal-corridor ratio decreased by 4.4%, producing a visibly fuller transverse smile (*d* = 0.66). Upper-lip length and inter-commissural width increased by about 1.0 mm and 1.6 mm, respectively (both *d* ≈ 0.5). Vertical smile dimensions, incisor display, and global facial height and width remained stable (|*d*|≤0.18). Measurement reliability ranged from good to excellent (ICC = 0.60–0.93(Intraclass Correlation Coefficient).

**Conclusion:**

Orthognathic surgery improved transverse smile fullness in patients with class III skeletal deformities while preserving vertical dental display, affirming the value of 3D assessment.

**Clinical relevance:**

Three-dimensional stereophotogrammetry offers objective, reproducible documentation of soft-tissue changes and should be integrated into routine orthognathic evaluations. Unlike previous studies relying on cephalometry and photographs, this is the first study to utilize 3D stereophotogrammetry for assessing smile parameters.

## Introduction

 The English botanist John Ray encapsulated the power of beauty and the centrality of smile aesthetics with the dictum, “Beauty is a power, and a beautiful smile is its sword” [1]. Among the various components of dentofacial harmony, the smile plays a crucial role in guiding modern treatment decisions [2].

Orthognathic procedures address two intertwined aims: rectifying functional impairments and refining facial appearance. Classic indications, such as compromised mastication, speech anomalies, or occlusal instability remain compelling reasons for intervention; however, concerns over disproportionate facial contours and disharmony are often equally influential. A substantial body of evidence shows that most candidates seek surgery to secure both functional recovery and restore anatomical balance and enhance facial aesthetics [3]. Modig et al. for example, reported that although 55% of patients cited functional improvement as their primary goal, 30% were primarily driven by aesthetic consideration [4].

In recent years, the drive to optimize smile aesthetics has become a major indication for orthognathic surgery. A substantial proportion of patients seek orthodontic intervention primarily to improve their smile, and smile analysis remains a core element of soft-tissue evaluation in contemporary orthodontics [5]. A patient’s smile must therefore be assessed not only by the patient, but also by the treating orthodontist when formulating a treatment plan. Beyond its role in facial aesthetic appraisal, the smile is pivotal to emotional wellbeing and social interaction [6].

The characteristics of a smile arise from the complex interaction between static (resting) and dynamic (functional) relationships of the dentoskeletal structures and the overlying soft tissues [7]. Malocclusions, especially class III discrepancies, exert a pronounced negative effect on both facial profile and smile aesthetics, making comprehensive smile analysis an indispensable step in orthodontic treatment planning [8].

Ackerman et al., using a neurophysiologic perspective, categorized the multitude of possible smiles into two principal types [9–11]. The social smile is the consciously controlled expression typically used in social settings or when posing for photographs [12]. Since it is reproducible and can be elicited on command, the social smile is routinely used in the assessment of facial aesthetics and smile characteristics [13]. In a stereophotogrammetric study by Dobreva et al. involving 93 young adults, repeated social smiles captured 4 weeks apart showed mean positional changes of the lips of less than 1.5 mm, underscoring the stability of this smile type [14].

In patients with class III deformities, skeletal disharmony compromises overall facial harmony and the perception of the smile [15, 16]. These patients frequently display impaired smile aesthetics that can diminish self-confidence and hinder social interactions; therefore, orthognathic surgery affords both functional correction and aesthetic benefit [17]. Individuals with class III deformities typically present with a concave profile, a retrusive nasomaxillary complex, and a protrusive lower lip. The maxillary arch is narrower than the mandibular counterpart, resulting in negative overjet and reduced overbite [18]. Maxillary incisors are usually buccally inclined, while mandibular incisors compensate lingually; the interlabial gap is increased and the smile arc tends to be inverted or flattened. Posterior smile corridors are wider, and the last visible posterior teeth are often canines or first premolars due to maxillary retrusion [8].

Zhong et al. evaluated preoperative and postoperative smile parameters in 37 patients with class III deformities and reported reduced upper-lip thickness, increased lower-lip thickness secondary to mandibular protrusion, a lower smile line, and a downward upper-lip curvature preoperatively [16]. Smile asymmetry is also common in this population and should be considered in orthodontic and surgical planning. Midfacial deficiency accentuates nasal prominence, adversely affecting profile both at rest and when smiling [19].

Accurate recording of facial morphology underpins diagnosis and treatment planning. Traditional assessments use 2D lateral cephalograms and extraoral photographs, but these modalities cannot fully capture the intricate dynamics of soft tissues [20, 21]. Driven by rising aesthetic expectations and advances in imaging, 3D techniques now permit more precise analyses and contribute to superior aesthetic outcomes. 3D photographic systems rapidly and non-invasively acquire surface anatomy and markedly expand quantitative analytical capabilities [22]. Although many studies of smile aesthetics use conventional photography [23], digital videography [24], and real-time biometric measurements, the inherent limitations of 2D methods are accentuated in class III malocclusion, where skeletal deformity profoundly influences facial aesthetics [25].

In orthognathic-surgical planning, Plooij et al. conceptualized the facial soft tissues, facial skeleton, and dentition as a triad with each element playing a decisive role in surgical decision making and in formulating treatment objectives [26]. Collectively, these considerations highlight the requirement for comprehensive, dynamic, and 3D-based evaluation of the smile (particularly in patients with class III deformities) to inform treatment strategies that harmonize functional correction with optimal aesthetic outcomes.

One of the most fundamental keys to success in orthognathic surgery is a meticulous and comprehensively executed treatment plan. The clinical contribution of this study is to emphasize the importance of evaluating not only skeletal harmony, but also aesthetic parameters (particularly smile aesthetics) within surgical limits and during the planning phase of orthognathic surgery. To this end, the integration of 3D stereophotogrammetry into the planning process aims to promote a more holistic approach to both aesthetic and functional goals, thereby enhancing the overall standards of surgical planning. The current study aimed to build a novel 3D superimposition technique and use 3D stereophotogrammetry to assess smile parameters in patients with skeletal class III deformities undergoing orthognathic surgery. By examining variables, such as lip height ratio, lip asymmetry, and lip area, this study aimed to provide a detailed and objective evaluation of soft tissue modifications associated with surgical correction. This investigation not only represents a significant step forward in capturing the nuances of soft tissue adaptation, but also marks the first study to use 3D stereophotogrammetry specifically for preoperative and postoperative smile analysis in patients with class III orthognathic deformities.

## Materials and methods

The study was approved by the Başkent University Institutional Review Board and Ethics committee (project no: D-KA24/11). All participants provided their written informed consent in accordance with the Declaration of Helsinki before participating. The included patients had class III deformities and were undergoing orthognathic surgery for moderate to severe malocclusion or dentofacial deformities necessitating surgical correction and had complete pretreatment and posttreatment data, such as lateral cephalometric and panoramic radiographs, extraoral and intraoral photographs, and 3D stereophotogrammetry taken in social smile and resting positions.

Retrospective data from 21 individuals (mean age 22.7 ± 3.0 years) who underwent Le Fort I surgery in combination with mandibular surgery between 2020, and 2023, at the Başkent University Oral and Maxillofacial Surgery department, were reviewed. Patients were included if they had complete preoperative and postoperative 3D smile images (using a 3D stereophotogrammetry system [3DMD, Atlanta, Georgia]) and corresponding cone-beam computed tomography (CBCT) scans. The average follow-up period from orthognathic surgery to postoperative data collection was approximately 8 months, ranging from − 1 to + 2 months postoperatively.

Exclusion criteria included individuals with facial deformities resulting from trauma or cleft lip-palate, craniofacial syndromes, a history of orthognathic surgery, psycho-organic disorders, behavioral disorders, systemic arthritis, or muscular disease. Patients with maxillary advancement outside the range of 2–8.5 mm or mandibular setback outside 2–5.5 mm were also excluded to ensure standardized skeletal corrections across the sample. Patients unable to comply with study protocols were also excluded. Patients without complete preoperative and postoperative 3D smile stereophotogrammetry and CBCT records were also excluded from the study.

Preoperative and postoperative CBCT images were taken with the patient sitting in an upright relaxed position with maximum interdigitation and with the lips at rest. No facial support was used to help obtain an unobstructed recording of soft tissue.

Images of patients smiling were captured preoperatively and 8 ± 2 months after surgery using a 3D stereophotogrammetry system. According to the guidelines of the camera system manufacturer, participants were seated on a chair positioned at a standardized distance (approximately 70 cm) from the camera, with their head slightly tilted upwards (approximately 10° to the horizontal plane). Patients were asked to stand still and smile showing their teeth during the photographs (i.e., social smile). All images were acquired by the same person for standardization purposes. The vertical and horizontal line markings were used for head orientation in 3D osteotomy planning. The surgical movements were determined according to the means in Arnett’s analysis of soft tissue with the constructed vertical line taken as the true vertical line reference.

**Fig. 1:**
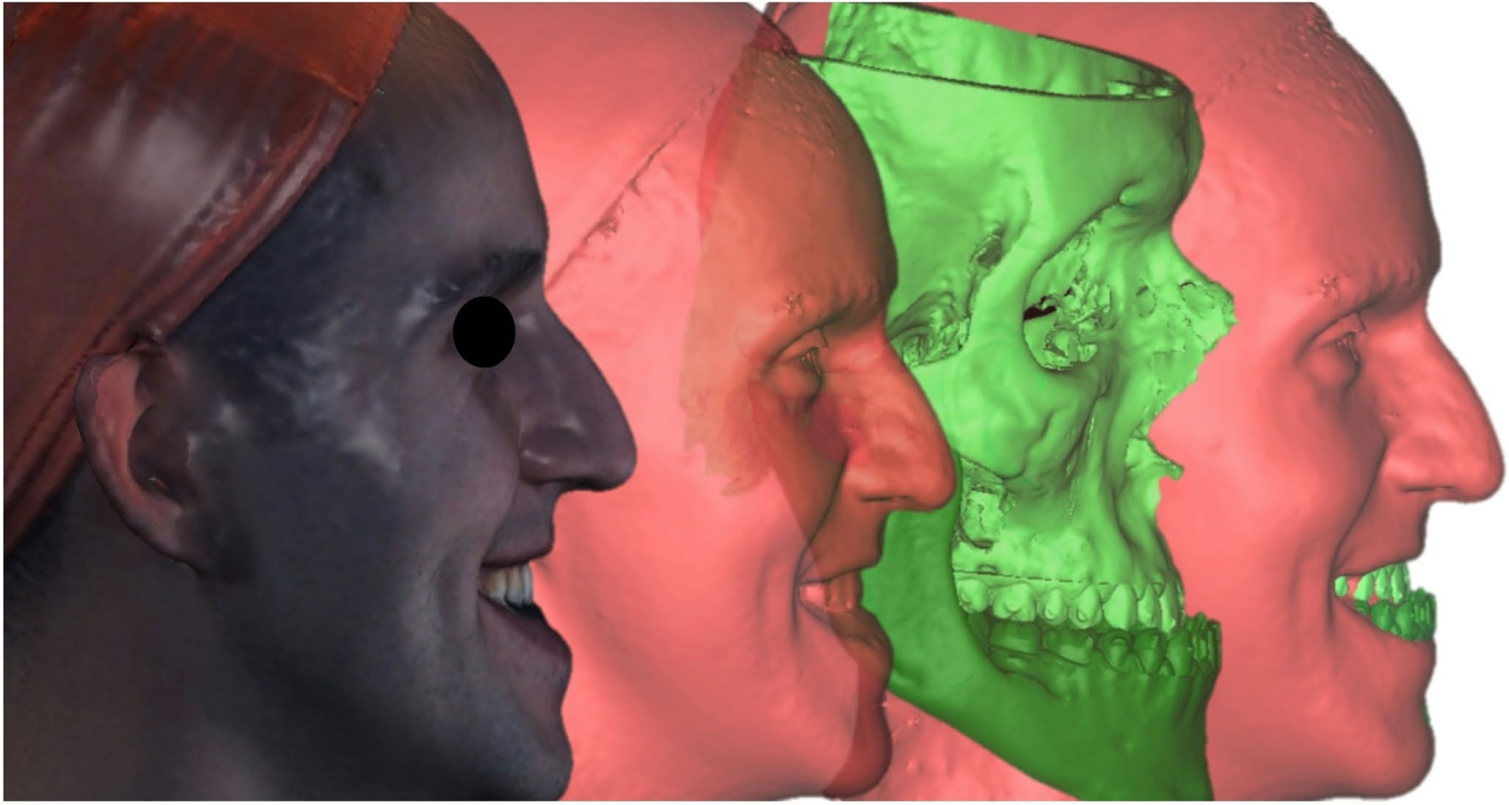
Three-modality fusion workflow sequential registration of posed-smile stereophotogrammetry(right), CBCT-derived bone and soft-tissue meshes(center), and high-resolution intraoral scans(left) combines all surfaces in a common coordinate system for standardized metric extraction. Multi-region best-fit alignment was performed on the nasal bridge, glabella, and maxillary incisor enamel to ensure accurate and reproducible superimposition across all datasets

CBCT data were processed with DICOM processing software (3D Slicer). The bone tissue and soft tissue were separately rendered and exported as stereolitography (STL) files. Intraoral scanning data were superimposed using a 3D inspection and mesh processing software (Gom-Inspect) and low-quality tooth surface data on the STL image derived from CBCT was replaced with high-quality intraoral scans. Facial scans, which were obtained during social smiles, were subsequently imported and registered to the CBCT-derived soft-tissue mesh using a multi-region best-fit protocol. To ensure reproducibility, the alignment was performed on expression-insensitive and geometrically stable regions, specifically the nasal bridge, soft-tissue glabella, and the visible surfaces of the maxillary central incisors. After sequentially merging all three datasets, a composite 3D model that included high-resolution facial structures during posed smile and tooth surfaces connected to 3D rendered bone tissue from CBCT image was obtained for standardized metric analysis (Fig. 1).

Sequential registration of posed-smile stereophotogrammetry, CBCT-derived bone and soft-tissue meshes, and high-resolution intraoral scans combines all surfaces in a common coordinate system for standardized metric extraction.

All surgical procedures were performed by the same experienced surgical team at the Başkent University Department of Oral and Maxillofacial Surgery, using standard Le Fort I osteotomy and bilateral sagittal split ramus osteotomy (BSSRO) techniques to ensure consistency in operative planning and execution throughout the study cohort.

### Smile and facial measurements

Quantitative soft tissue analysis was performed using 3D smile images acquired via stereophotogrammetry, which were spatially registered with the corresponding CBCT datasets. This superimposition enabled the alignment of both hard and soft tissue landmarks within a common coordinate system, allowing for standardized and reproducible measurements along defined anatomical axes.

**Fig. 2:**
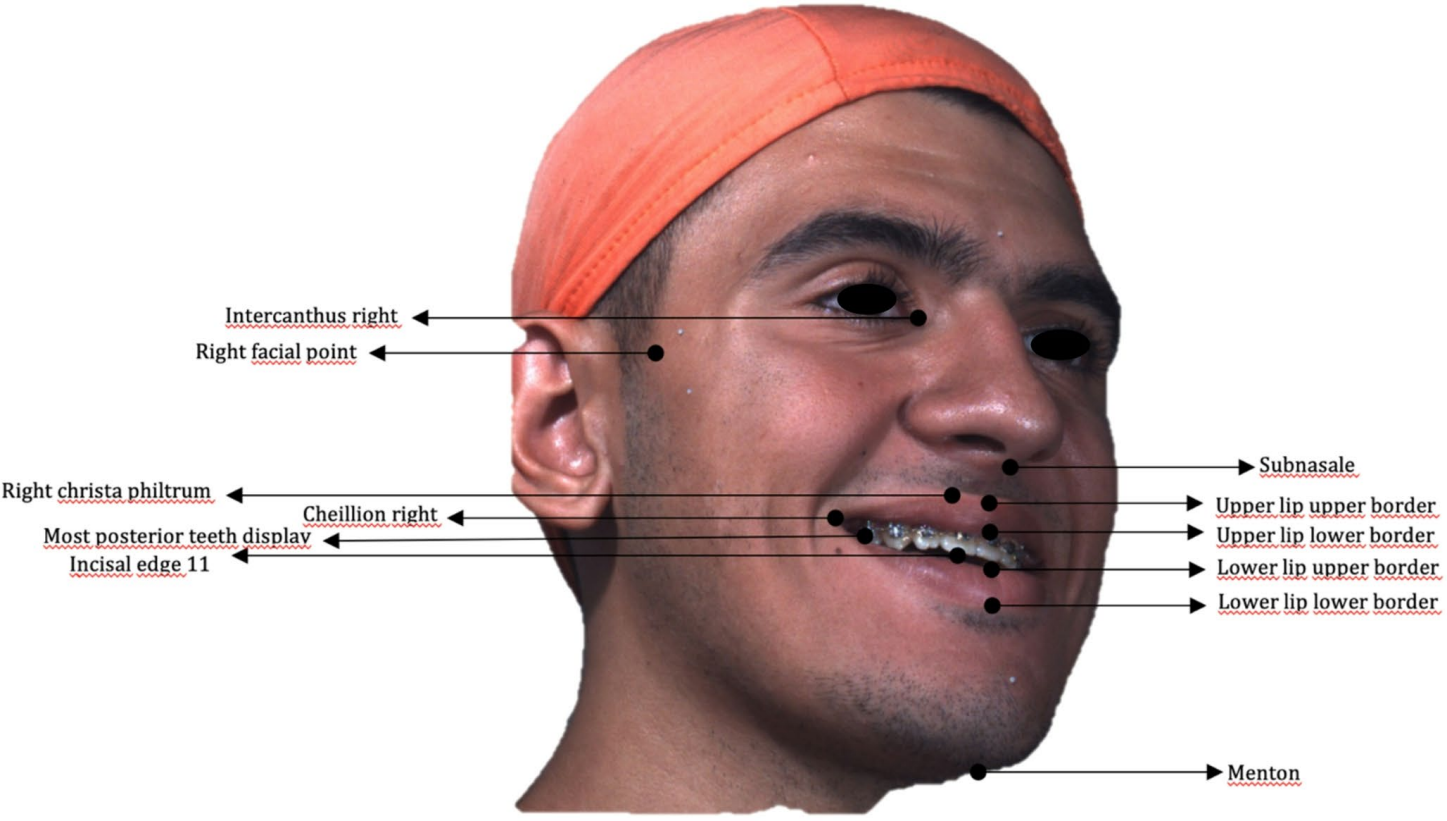
Posed-smile 3D scan illustrating the facial and dental landmarks (e.g., intercanthus, philtrum, cheilion, lip borders, incisal edge, menton, etc.) used for soft-tissue and smile measurements

3D soft-tissue analysis was conducted using preoperative and postoperative 3D stereophotogrammetric images spatially registered to CBCT datasets. The fusion of these two imaging modalities enabled the construction of an anatomically consistent reference system based on cranial landmarks. Cephalometric landmarks were delineated on the basis of these superimpositions (Tables 1 and 2) (Fig. 2). The sagittal plane was defined by the Nasion–Sella–ANS (anterior nasal spine) line, while the horizontal reference plane was established using the Frankfurt horizontal plane (defined by bilateral Porion and Orbitale points: PoR, PoL, OrR, and OrL). The midfacial vertical axis was drawn from point MP, located at the midpoint between the eyebrows, to menton.

**Table 1:** Soft-tissue facial landmarks; abbreviations and anatomical definitions used for the 3D smile analysis

Soft-tissue landmark	Abbreviation	Definition
Right cheilion	Ch R	Right oral commissure (corner of the mouth)
Left cheilion	Ch L	Left oral commissure
Right crista philtri	Chp R	Point on the right lateral border of the philtrum, immediately superior to the upper vermilion margin
Left crista philtri	Chp L	Point on the left lateral border of the philtrum, immediately superior to the upper vermilion margin
Subnasale	Sn	Deepest midline point at the columellar base, where the nasal septum meets the upper lip
Vermilion border	–	Junction line between lip vermilion and facial skin
Labiale superius	Ls	Midpoint of the upper-lip vermilion border
Labiale inferius	Li	Midpoint of the lower-lip vermilion border
Upper and lower stomion	St	Midpoint where upper and lower lips contact on the facial midline
Right endocanthion	En R	Medial canthus of the right palpebral fissure (meeting point of upper and lower eyelids)
Left endocanthion	En L	Medial canthus of the left palpebral fissure
Lateral facial soft-tissue point	–	Most lateral soft-tissue prominence on each side at zygomatic level

**Table 2:** Hard-tissue facial landmarks; abbreviations and anatomical definitions used for the 3D smile analysis

Hard-tissue landmark	Abbreviation	Definition
Incisal edge of tooth 11	–	Cutting edge of the maxillary right central incisor
Posterior visible teeth	–	Most posterior teeth visible during posed smile (right and left arches)
Nasion	N	Midline point on the nasal bridge along the line connecting the endocanthia
Sella	S	Centre of the sella turcica
Anterior nasal spine	ANS	Midline bony projection at the base of the nasal cavity
Menton	Me	Lowest midline point on the bony chin

### Measurements derived from the selected landmarks

1. Lip morphology parameters
  Lip length: linear distance from subnasale (Sn) to upper stomion (St); expresses vertical length of the upper lip [27]
  Lip width: horizontal distance between right and left cheilion [28]
  Philtrum width: distance between right and left crista philtri; shows basal width of the philtrum [28]
  Upper-lip and lower-lip thickness: vertical distance between upper and lower vermilion borders at the midline; represents soft-tissue bulk of each lip [29]
  Cupid’s bow angle: angle formed by the two crista philtri and labiale superius; quantifies Cupid’s bow curvature (Fig. 3) [30]

**Fig. 3:**
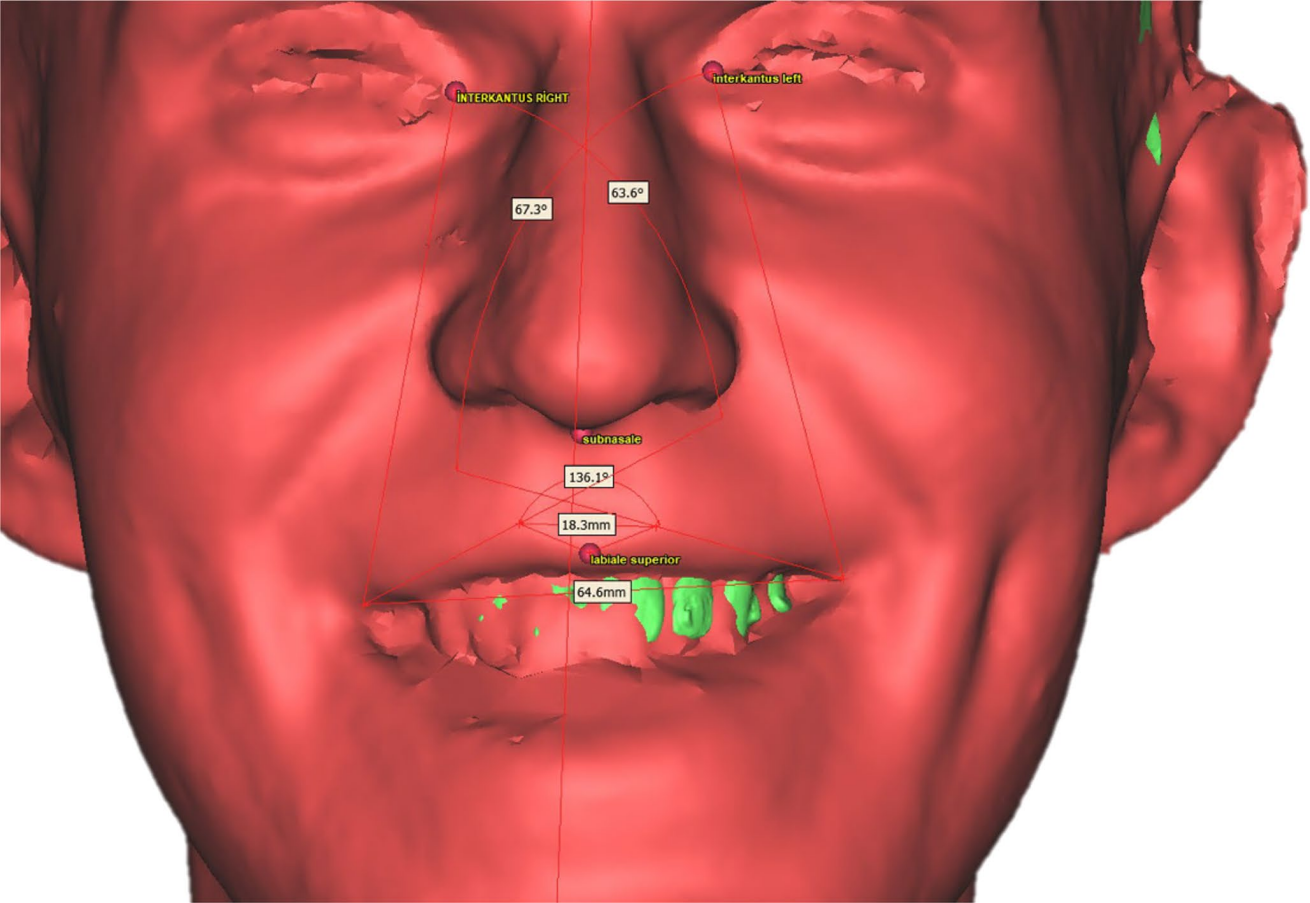
3D facial model showing the automated angular metrics: lip angle, cupid’s bow angle, nasolabial angle, and maxillary incisor display used in quantitative smile analysis
  Lip angle: angle between endocanthion–cheilion–philtrum points; reflects commissure position and smile line (Fig. 3) [28]
2. Smile parameters
  Smile width: distance between right and left cheilion during a posed smile [31]
  Lip-to-incisor distance: vertical distance from the incisal edge of tooth 11 to the upper-lip border [32]
  Smile height: vertical distance between upper and lower stomion; depicts lip separation during smiling [11]
  Buccal-corridor ratio: (right + left posterior space between last visible tooth and commissure)/inter-commissural width × 100; indicates lateral dark space contribution during smiling [33]
  Smile index: ratio of smile width-to-smile height; represents horizontal-to-vertical aesthetic balance of the smile [34]
3. Facial dimensions
  Facial height: linear distance from Mid-Brow point (MP) to Menton; measures vertical facial dimension
  Facial width: Horizontal distance between right and left lateral facial soft-tissue points; used for transverse facial assessment [35]

All measurements were performed within aligned 3D datasets following standard protocols. Each metric was recorded twice by a standardized examiner to ensure intra-observer reliability. 

### Statistical analysis

All statistical analyses were performed with NCSS version 2023 (NCSS LLC, Kaysville, UT, USA). Descriptive results were reported as mean ± standard deviation (SD). Intra-observer repeatability was first verified by re-landmarking ten randomly selected 3D datasets 1 month after the initial session; the two-way mixed-effects intraclass correlation coefficient—ICC (2, 1)—classified reliability as poor (< 0.50), moderate (0.50–0.75), good (0.75–0.90), or excellent (> 0.90). Normality of preoperative to postoperative differences (T1–T0) was examined with the Shapiro–Wilk test. Variables that reached normality were compared by paired-sample *t*-tests, whereas non-normal data were analyzed with Wilcoxon signed-rank tests. Because 21 outcome variables were interrogated, raw *p*-values were adjusted with the Benjamini–Hochberg false discovery rate (FDR) procedure and FDR-corrected values < 0.05 were interpreted as statistically significant.

Beyond hypothesis testing, each pre-post change was expressed as Cohen’s *d* for paired samples. We classified Cohen’s conventional thresholds as negligible (< 0.20), small (0.20–0.49), medium (0.50–0.79), and large (≥ 0.80); since *d* conveys clinical relevance independent of sample size. This dual emphasis on FDR-adjusted significance and Cohen’s *d* allowed us to distinguish changes that were detectable from those likely to be perceptible in practice; for example, a medium *d* (≈ 0.5) was interpreted as a shift that most clinicians and patients could notice even if the FDR-corrected *p* did not reach 0.05. In summary, statistical inference in this study rested on three pillars: measurement reliability (ICC), error-controlled significance testing (FDR), and magnitude-based interpretation (Cohen’s *d*) to provide a balanced evaluation of smile transformation in patients with class III deformities following orthognathic surgery.

Due to the retrospective nature of the study and the use of existing patient records meeting the inclusion criteria, it was not feasible to perform a formal a priori power analysis based on expected effect sizes for the primary outcomes. Therefore, a post-hoc power analysis was conducted using G*Power to evaluate the adequacy of the sample size. Assuming a significance level of 0.05 and a power of 90%, a minimum of 21 patients was estimated to be sufficient to detect an effect size of 0.7.

## Results

21 subjects were included in this study of which nine were females and 12 were male. The mean age of the participants was 21.67 ± 2.74 years preoperatively and 22.48 ± 2.73 years when the postoperative data were collected (mean ± SD). The average follow-up period from orthognathic surgery to the acquisition of postoperative data was approximately 8 months (range 6–9 months; Table 3).

**Table 3:** Descriptive statistics for participant age (baseline T0 and follow-up T1, stratified by sex) and planned skeletal movements of the maxilla and mandible in sagittal and vertical planes (mean ± SD and range)

Variable	Subgroup	Mean ± SD	Maximum	Minimum
Age T0 (years)	Total	21.67 ± 2.74	27.00	17.00
	Male	22.33 ± 3.08	27.00	17.00
	Female	20.78 ± 2.05	25.00	18.00
Age T1 (years)	Total	22.48 ± 2.73	28.00	18.00
	Male	23.08 ± 3.06	28.00	18.00
	Female	21.67 ± 2.12	26.00	19.00
Maxilla–sagittal (mm)	Total	4.81 ± 2.39	8.50	2.00
Maxilla–vertical (mm)	Total	0.17 ± 2.57	6.50	–5.50
Mandible–sagittal (mm)	Total	–3.89 ± 1.67	−2.20	–5.50
Mandible–vertical (mm)	Total	0.28 ± 2.27	4.00	–4.00

*SD* standard deviation

**Fig. 4:**
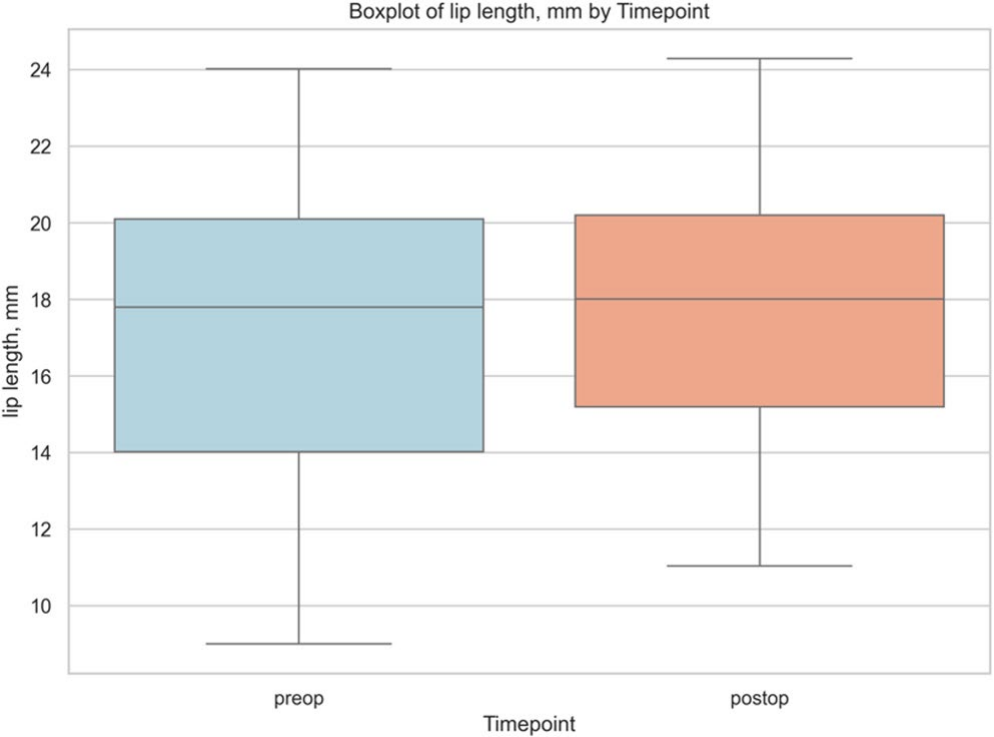
Box and whisker plot of upper-lip length (Sn–St) at baseline (preoperative) and 6-month follow-up (postoperative), illustrating a modest median increase (~ 1 mm) with overlapping interquartile ranges

Postoperative analysis revealed an ≈ 1 mm increase in upper-lip length (16.96 ± 4.17 mm vs. 17.95 ± 3.79 mm; Δ=+0.98 mm, *d* = + 0.51), implying a slight inferior shift of the vermilion border. Although the FDR-adjusted *p* did not reach significance, the medium effect size and excellent reliability (ICC = 0.863) indicates a clinically perceptible change most plausibly attributable to either deliberate inferior repositioning or counterclockwise rotation of the maxilla or an anterior impaction that was smaller than the posterior, resulting in a net downward soft-tissue displacement (Fig. 4).

Preoperative and postoperative linear and angular lip metrics are summarized in Table 4. None of the variables retained statistical significance after Benjamini–Hochberg correction; however, two dimensions displayed medium magnitude effect sizes (|*d*|≈0.5) that might still be clinically noticeable.

**Table 4:** Pre-operative versus 6-month postoperative soft-tissue metrics with paired differences (Δ), FDR-adjusted *p*-values, and effect sizes (Cohen’s *d*)

Metric	Preoperative (mean ± SD)	Postoperative (mean ± SD)	Δ (postoperative– preoperative)	FDR-adjusted *p*	Cohen’s d
Lip length (Sn–St, mm)	16.96 ± 4.17	17.95 ± 3.79	**+ 0.98**	0.2003	**+ 0.51**
Inter-commissural width (mm)	59.99 ± 4.44	61.59 ± 2.85	**+ 1.60**	0.1571	**+ 0.51**
Lip width (vermilion border, mm)	64.15 ± 4.44	62.69 ± 9.49	–1.46	1.0000	–0.16
Philtrum width (mm)	14.73 ± 3.26	15.36 ± 3.18	+ 0.63	0.7386	+ 0.30
Upper-lip thickness (mm)	6.95 ± 1.48	8.33 ± 8.82	+ 1.38	0.7386	+ 0.15
Lower-lip thickness (mm)	7.35 ± 1.41	6.78 ± 1.63	–0.57	0.7386	–0.30
Cupid’s bow angle (mm)	146.10 ± 9.98°	142.47 ± 10.39°	–3.63°	0.7386	–0.33
Right lip angle	65.62 ± 6.94°	67.09 ± 4.56°	+ 1.47°	0.7386	+ 0.22
Left lip angle	67.09 ± 6.23°	68.50 ± 5.06°	+ 1.40°	0.7386	+ 0.21
Chelion right (distance to mid-sagittal, mm)	30.81 ± 3.32	30.04 ± 4.71	–0.77	0.8434	–0.17
Chelion left (distance to mid-sagittal)	32.35 ± 3.54	33.67 ± 5.13	+ 1.32	0.7386	+ 0.28
*FDR* false discovery rate. *p* < 0.05, cohen’s d: Negligible): |d| < 0.20, small: 0.20 ≤ |d| < 0.50, medium: 0.50 ≤ |d| < 0.80, large: |d| ≥ 0.80					

**Fig. 5:**
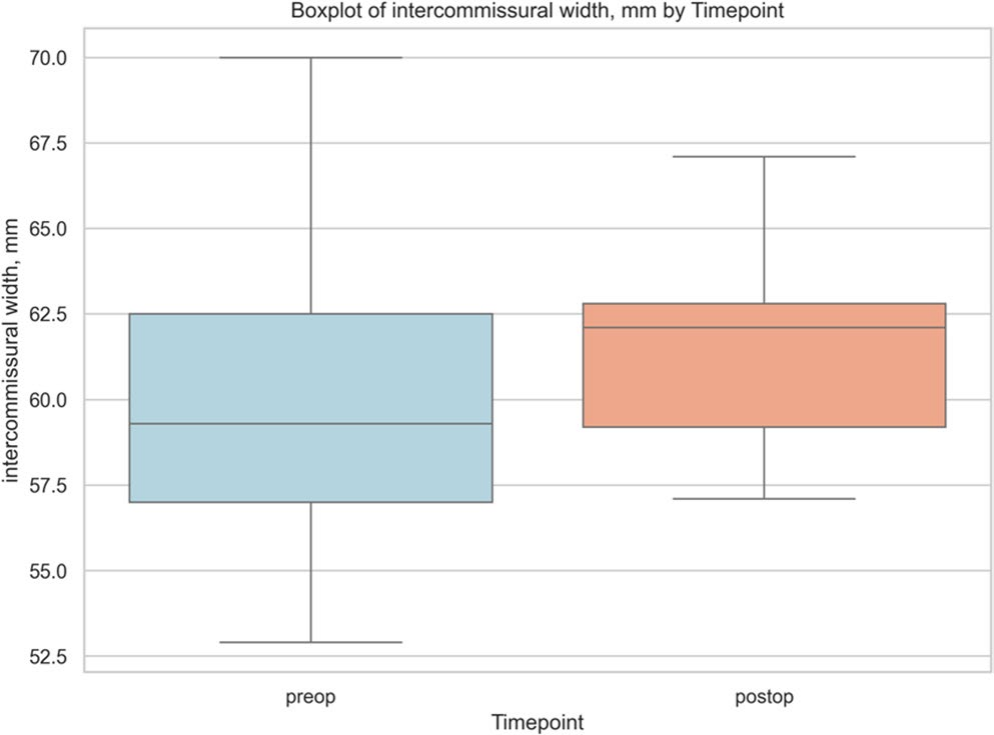
Box and whisker plot of inter-commissural width at baseline (preoperative) and 6-month follow-up (postoperative), showing a slight postoperative widening with largely overlapping interquartile ranges

Inter-commissural width widened in parallel by about 1.6 mm, yielding the same medium effect size (*d* = + 0.51). While this finding did not reach statistical significance after correction, its moderate reliability (ICC = 0.597) suggests a genuine, if subtle, lateral displacement of the mouth corners that was consistent with the skeletal advancement achieved (Fig. 5).

Upper-lip and lower-lip thickness changes were small and opposite in direction: the upper lip became slightly thicker (+ 1.38 mm), whereas the lower lip thinned (− 0.57 mm), implying that volumetric alterations remained minimal within the follow-up period.

Angular measures showed similar minor shifts. The Cupid’s bow angle decreased by 3.6°, indicating a subtle sharpening of the arc. Right and left lip angles rotated upward by approximately 1.4° (|*d*|≈0.22), changes that were below thresholds typically perceived by observers.

Lastly, commissural position relative to the mid-sagittal plane remained essentially unchanged: the right cheilion moved medially by 0.8 mm, while the left shifted laterally by 1.3 mm, neither alteration reaching statistical or clinical relevance (|*d*|≤0.28).

In summary, after bimaxillary correction, lip morphology was largely preserved. The only clinically noteworthy tendencies were a moderate lengthening of the upper lip and a parallel widening of the inter-commissural distance, which both aligns with the intended inferior and lateral repositioning of the soft tissues, while all other dimensions and angles had minimal or negligible change. Although no static lip metric survived multiple-testing correction, the medium effect sizes for upper-lip length and inter-commissural width suggest that patients could perceive a subtly longer, slightly broader smile at rest. These findings, coupled with good measurement reliability, can inform patient counselling and set realistic expectations for soft-tissue outcomes following bimaxillary correction in cases of class III deformities.

**Fig. 6:**
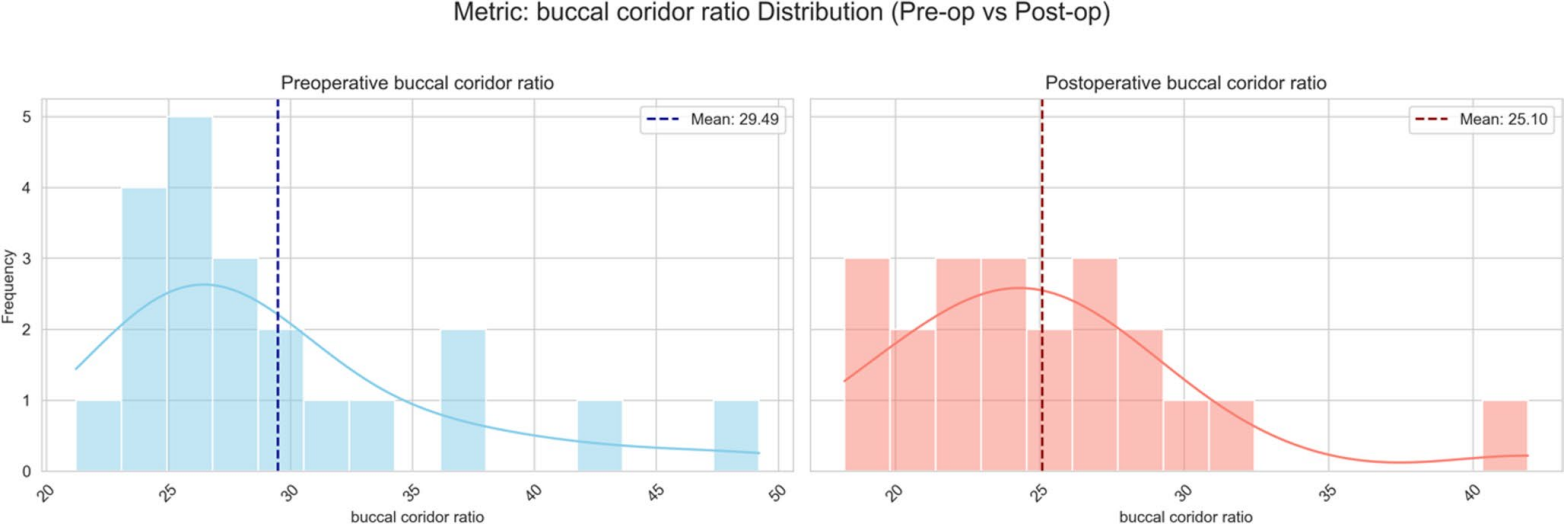
Histograms with density curves of the buccal-corridor ratio preoperatively (left) and 6 postoperatively (right), illustrating a postoperative leftward shift and narrowing that indicate reduced transverse dark space during smiling

The buccal-corridor ratio decreased markedly (from 29.5 ± 7.0% to 25.1 ± 5.4%) and was the only parameter that remained significant after Benjamini–Hochberg correction. The change carried a medium effect size (*d*=–0.66) and produced a visibly fuller smile by reducing the lateral dark spaces. The low ICC (0.356), observed for the buccal-corridor ratio likely reflects the inherent variability of this parameter. Because the measurement depends on identifying the last visible posterior tooth, even subtle differences in smile intensity can alter posterior tooth visibility and reduce repeatability. In addition, the depth-dependent nature of 3D stereophotogrammetry makes distinguishing the most posterior tooth challenging, as minor variations between the photographic and digital artificial lighting situations might have caused a difference in perception of buccal corridors. Although the ICC for this metric was low the buccal corridor improvement represents a meaningful aesthetic enhancement and should be highlighted to patients with class III deformities as a predictable benefit of bimaxillary correction (Fig. 6). Therefore, despite the statistically significant reduction observed postoperatively, this finding should be interpreted cautiously in light of its lower reproducibility.

Sex-based subgroup analysis identified significant differences in three transverse smile parameters. Females exhibited a greater postoperative increase in intercommissural width compared with males (females: +3.22 mm; males: +1.39 mm; *p* = 0.030, *r* = 0.48). In addition, the reduction in buccal corridor ratio was more pronounced in females. No other variable demonstrated significant sex-related differences.

Lip-to-incisor distance showed minimal change (7.59 ± 2.50 mm → 8.01 ± 1.87 mm); with poor repeatability (ICC = 0.283). Incisal display remained essentially stable (Table 5).

**Table 5:** Preoperative versus 6-month postoperative dynamic-smile metrics—buccal-corridor ratio, lip-to-incisor distance, smile height, and smile index—with paired change (Δ), FDR-adjusted *p*-values, and effect sizes (Cohen’s *d*)

Metric	Preoperative (mean ± SD)	Postoperative (mean ± SD)	Δ (postoperative–preoperative)	FDR-adjusted *p*	Cohen’s d
Buccal-corridor ratio (%)	29.49 ± 6.98	25.10 ± 5.37	**−4.39%**	**0.0107**	**−0.66**
Lip–incisor distance (mm)	7.59 ± 2.50	8.01 ± 1.87	+ 0.42 mm	0.9254	+ 0.16
Smile height (mm)	13.05 ± 3.64	12.60 ± 2.62	−0.45 mm	0.7400	−0.13
Smile index (width/height)	5.36 ± 1.59	5.57 ± 1.39	+ 0.21	0.7400	+ 0.16

*FDR* false discovery rate, *SD* standard deviation

For postoperative evaluation, mean smile height exhibited a negligible reduction of 0.45 mm (13.05 ± 3.64 mm preoperative vs. 12.60 ± 2.62 mm postoperative). The change in smile height was minimal and the measurement was only moderately reliable (ICC = 0.409), indicating that vertical lip opening did not change after surgery. Likewise, smile height and the lip-to-incisor distance remained almost the same, showing that the maxillary movements improved aesthetics without reducing how much of the teeth were shown (Table 5).

Similarly, the smile index (transverse-to-vertical ratio) increased marginally from 5.36 to 5.57. With moderate repeatability (ICC = 0.602), this minimal shift suggests an imperceptible tendency toward a proportionally wider smile frame, carrying no clinically consequential effect on smile aesthetics (Table 5).

Mean face height decreased by only 1.07 mm (136.36 ± 10.08 137.43 ± 10.62 mm preoperative, 136.36 ± 10.08 postoperative), a difference within the range of normal soft-tissue variation and supported by good measurement reliability (ICC = 0.843). Bizygomatic width remained essentially stable (146.2 ± 6.05 mm vs. 146.20 ± 5.73 mm; Δ = 0.07 mm). These findings show that bimaxillary surgery balanced the sagittal profile without producing noticeable changes in overall facial height or width (Table 6).

**Table 6:** Preoperative versus 6-month postoperative global facial dimensions—brow midpoint-to-menton height and bizygomatic width—with paired change (Δ), FDR-adjusted *p*-values, effect sizes (Cohen’s *d*), and ICC (2, 1) for measurement reliability

Metric	Preoperative (mean ± SD)	Postoperative (mean ± SD)	Δ (postoperative–preoperative)	FDR-adjusted *p*	Cohen’s d	ICC (2, 1)
Face height (brow midpoint-menton, mm)	137.43 ± 10.62	136.36 ± 10.08	–1.07	0.7386	–0.18 (negligible)	0.843 (good)
Face width (bizygomatic soft tissue, mm)	146.28 ± 6.05	146.20 ± 5.73	–0.07	0.9254	–0.03 (negligible)	0.933 (excellent)

*FDR* false discovery rate, *ICC* intra-class correlation coefficient

## Discussion

The stereophotogrammetric analysis in our study provides a 3D perspective on smile changes after orthognathic surgery for patients with class III deformities. The reproducibility of the social smile and the high measurement reliability of the 3dMD system further strengthen the validity of our findings. The acquisition protocol used in this study has been shown to produce consistent smile morphology across repeated captures. Overall, we found that transverse smile parameter improved and there was a notably marked reduction in buccal corridor space, whereas vertical smile dimensions and soft-tissue curvature showed minimal change. Both upper-lip length and inter-commissural width did not meet the corrected threshold significance, yet each showed a medium effect size indicative of clinically appreciable lengthening. Given the limited sample size and the loss of power inherent to multiplicity adjustment, these findings suggest that, although not statistically verified, the observed increase in lip length and mouth width are likely to be perceived by patients and, therefore, warrant clinical consideration. However, key aesthetic elements, such as the interlabial gap (smile height) and smile index (width-to-height ratio), remained largely stable on average. These findings suggest that while jaw repositioning can enhance certain aspects of the smile (especially width and fullness), many frontal smile characteristics are inherently resilient to surgical change or require more time to adapt. Our results both align with and diverge from previous studies, warranting a detailed comparison with the existing literature. Compared with previous 2D photographic studies, the 3D stereophotogrammetric workflow adopted in the present analysis provides several methodological advantages. Three-dimensional imaging enables true spatial measurement of soft-tissue displacement in all axes, reduces projection errors inherent to 2D methods. Furthermore, surface-based registration allows more precise superimposition of pre- and postoperative facial meshes, capturing subtle morphological changes that may not be detectable with traditional 2D evaluations.

### Lip morphology (philtrum height and lip form)

Orthognathic correction produced subtle alterations in lip morphology. In our cohort, the upper lip lengthened by approximately 1 mm on average, post-surgery. This finding might reflect inferior repositioning or counterclockwise rotation of the maxilla, whereby the skeletal segment and the attached vermilion border migrate downward.

The ≈ 1 mm increase observed in our series was support with the clinical investigation, which was made to assess the upper lip changes after Le Fort 1 osteotomy for maxillary advancement and or impaction reported by Tahmasbi et al. In their study, they concluded that in the maxillary advancement group, the upper lip length increased by 2.23 ± 1 mm while in the maxillary advancement and impaction group, the lip length increased by 1.64 ± 0.30 mm [36]. This increase also falls within the − 0.8 mm to + 2.48 mm range summarized in the systematic review, confirming that our finding corroborates with the published literature [37].

Our findings are consistent with the work of Marşan et al. who analyzed 44 Turkish females with class III deformities who had bimaxillary surgery and reported that preoperatively, the upper lip length was 22.9 mm and 24.1 mm postoperatively. They also stated that counterclockwise rotation, typical in class III correction, can lengthen the upper lip by ~ 1 mm, parallel to our dataset [38]. In contrast, Louis et al. found no significant change in lip length after maxillary advancement procedure and highlights a stabilized lip length measurement [39].

Apart from length, the morphological shape of the lips—eg, the Cupid’s bow and commissure angles—remained stable. We found no significant alteration in the Cupid’s bow angle or in the angular orientation of the lip corners after surgery. This finding agrees with Dilaver et al., who reported that parameters, such as Cupid’s bow angle, lip width, and commissure form did not change appreciably following Le Fort I osteotomy [28].

The preservation of overall lip form is clinically relevant, as it indicates that functional and aesthetic improvements can be achieved without compromising the patient’s natural lip characteristics. However, for patients seeking substantial changes in lip contour or definition, adjunctive cosmetic procedures might still be required.

### Smile width and buccal corridor

One of the improvements we noted was in inter-commissural smile width and the buccal corridor. The inter-commissural width—the distance between the lip corners during a full smile—increased significantly after surgery (from ~ 60 mm to ~ 61.6 mm on average). Although a 1.6 mm increase (about 2.7%) is moderate, it suggests that patients could smile slightly broader post-treatment. This broader smile could result from alleviation of soft-tissue restrictions once the jaws are properly aligned.

Our result aligns with the findings of Dilaver et al. who also documented a significant decrease in buccal corridor area after orthognathic surgery. In their patients, especially those who underwent maxillary impaction, the lateral negative space of the smile diminished [28].

The postoperative decrease in buccal-corridor ratio appears to stem from both maxillary repositioning and the resulting transverse dentoalveolar display. When the maxilla is advanced the dental arch shifts anterior-laterally, bringing the posterior buccal segments closer to the commissures and exposing more premolar–molar teeth during smiling. This skeletal realignment reduces the negative lateral space that is characteristic of untreated maxillary retrusion [16].

In patients with skeletal class III deformities, maxillary retrusion typically places the dental arch further posterior to the oral commissures, exaggerating buccal corridor width. Surgical correction realigns the arch closer to the commissures, minimizing lateral shadowing and enhancing smile fullness. Furthermore, as Moore et al. emphasized, minimizing buccal corridor space enhances smile aesthetics by increasing transverse dental display [40]. Our findings support this view, demonstrating that forward repositioning of the maxilla not only corrects sagittal discrepancies, but also contributes to more balanced smile proportions.

### Smile index and interlabial gap (vertical smile dimensions)

The smile index, defined as the ratio of smile width to height, is commonly used to assess smile proportion. In our study, the smile index showed a minimal, non-significant increase after surgery (~ 5.36 to 5.57), indicating a preserved balance between transverse and vertical smile dimensions. Although smile width increased significantly, the slight reduction in interlabial gap (~ 0.45 mm) likely offset this effect. These findings are in line with Farzanegan et al. who also reported no significant postoperative changes to smile index or vertical smile height in patients with class III deformities [6]. The stability of interlabial gap might reflect individual neuromuscular patterns that are not easily altered by surgery alone. Chiang et al. on the other hand, reported a significant increase in smile index and a strong negative correlation between interlabial gap and smile index. Their use of 2D methods and larger sample size might account for this difference [5].

### Smile line and dental display

The smile line, reflecting the extent of incisal and gingival display when smiling, is a crucial determinant of smile aesthetics. For class III malocclusion, vertical maxillary excess or mandibular prognathism can result in disproportionate gingival or lower incisor exposure. In our study, despite the inclusion of patients who underwent both anteroposterior and vertical surgical repositioning, the vertical lip-to-incisor distance remained statistically unchanged postoperatively, indicating preservation of the smile line.

This finding aligns with Farzanegan et al., who similarly observed no significant difference in gingival or incisal display following class III correction [6]. However, Khamashata et al. reported a mean 3.3 mm maxillary advancement produced modest but desirable increases in maxillary central-incisor exposure, which was ≈ 0.5 mm at rest and 1 mm on smiling, without over-displaying gingiva. They also stated that the amount of tooth display was partly governed by upper-lip thickness and the use of cinch/V-Y techniques, underlining the importance of soft-tissue management in predicting final dental display [41].

The lack of significant change in our study could be attributed to individual soft-tissue variability and muscle adaptation. These findings emphasize that while orthognathic surgery can influence dental display when indicated, it does not inherently disrupt existing aesthetic harmony. Additional soft-tissue or periodontal interventions might still be required for patients with excessive gingival show or low smile lines.

### Smile symmetry and dynamics

Despite successful skeletal correction, our study found no statistically significant improvement in smile symmetry, postoperatively. The vertical alignment of the lip commissures and angular orientation of the smile line remained unchanged, consistent with findings by Kang et al. [42], who noted that improvements in skeletal symmetry do not always translate into more symmetrical smiles. They observed that while occlusal cants and chin deviations could be corrected, smile line asymmetry often persisted postoperatively, likely due to neuromuscular and soft-tissue influences.

Similarly, Chiang et al. reported limited changes in smile symmetry despite improved hard-tissue balance, noting that asymmetry around the mouth corners often remained unchanged. Several mechanisms could explain this: neuromuscular memory might preserve preoperative asymmetrical muscle activation patterns, soft-tissue lag or elasticity might undermine skeletal correction, or subtle residual asymmetries in the dentition or perioral musculature might persist post-surgically [5].

In the context of our cohort, the absence of measurable improvement in smile symmetry may similarly reflect the persistence of preoperative neuromuscular recruitment patterns and the time-dependent nature of perioral soft-tissue reorganization following orthognathic surgery within the 8-month postoperative window. Neuromuscular coordination and soft-tissue remodeling may continue beyond this timeframe; therefore, longer-term follow-up could potentially reveal additional adaptations, particularly in smile symmetry and perioral muscle balance.

Kang et al. and others have emphasized the need for realistic patient counseling, as smile symmetry might not improve without additional interventions, such as muscle retraining or soft-tissue refinement. Overall, our findings reinforce the literature consensus that while orthognathic surgery improves skeletal and occlusal symmetry, smile symmetry, especially in dynamic expression, could remain resistant to change in the short to medium term [40].

This study has several limitations that should be acknowledged. First, although three-modality fusion provides a comprehensive representation of skeletal and soft-tissue relationships, the superimposition of dynamic smile images onto CBCT-derived meshes may still introduce minor registration variability, particularly in regions influenced by expression. Second, the sample size was relatively small, which may have limited the statistical power to detect changes in parameters with moderate effect sizes. Finally, patients exhibited heterogeneous surgical movements, preventing subgroup analyses based on movement patterns; larger cohorts may allow such comparisons in future work.

## Conclusion

One of the unique contributions of this study is the use of 3D stereophotogrammetric imaging for smile analysis. While conventional 2D photographs and cephalometric evaluations often fall short in capturing the complexity of soft tissue morphology, 3D imaging techniques enable a detailed and objective assessment, offering considerable advantages for both surgical planning and patient counseling. In particular, the evaluation of dynamic facial expressions, such as the social smile, is greatly enhanced by 3D analysis, allowing for more accurate and standardized documentation of aesthetic changes before and after treatment. The structured 3D assessment used in this study holds clear clinical value in routine practice by enhancing diagnostic precision and patient communication. In conclusion, this study provides a multidimensional perspective on the effects of orthognathic surgery on smile aesthetics, contributing valuable insights into both clinical decision making and the management of patient expectations. For a comprehensive and reliable analysis of smile dynamics, 3D stereophotogrammetry should be considered the standard evaluation tool in contemporary maxillofacial surgery practice.

## Data Availability

No datasets were generated or analysed during the current study.
